# Analysis of Orthologous *SECONDARY WALL-ASSOCIATED NAC DOMAIN1 (SND1)* Promotor Activity in Herbaceous and Woody Angiosperms

**DOI:** 10.3390/ijms20184623

**Published:** 2019-09-18

**Authors:** Libert B. Tonfack, Steven G. Hussey, Adri Veale, Alexander A. Myburg, Eshchar Mizrachi

**Affiliations:** 1 Plant Physiology and Improvement Unit, Laboratory of Biotechnology and Environment, Department of Plant Biology, University of Yaoundé I, Yaoundé 0812, Cameroon; btonfack@uy1.uninet.cm; 2 Department of Biochemistry, Genetics and Microbiology, Forestry and Agricultural Biotechnology Institute (FABI), Genomics Research Institute (GRI), University of Pretoria, Pretoria 0002, South Africa; steven.hussey@fabi.up.ac.za (S.G.H.); adri.veale@fabi.up.ac.za (A.V.); zander.myburg@fabi.up.ac.za (A.A.M.)

**Keywords:** *SND1*, secondary cell wall, transcriptional regulation, vascular evolution

## Abstract

*SECONDARY WALL-ASSOCIATED NAC DOMAIN1* (*SND1*) is a master regulator of fibre secondary wall deposition in *Arabidopsis thaliana* (Arabidopsis), with homologs in other angiosperms and gymnosperms. However, it is poorly understood to what extent the fibre-specific regulation of the *SND1* promoter, and that of its orthologs, is conserved between diverged herbaceous and woody lineages. We performed a reciprocal reporter gene analysis of orthologous *SND1* promoters from Arabidopsis (*AthSND1*), *Eucalyptus grandis* (*EgrNAC61*) and *Populus alba* × *P. grandidentata* (*PagWND1A*) relative to secondary cell wall-specific *Cellulose Synthase4 (CesA4*) and *CesA7* promoters, in both a non-woody (Arabidopsis) and a woody (poplar) system. β-glucuronidase (GUS) reporter analysis in Arabidopsis showed that the *SND1* promoter was active in vascular tissues as previously reported and showed interfascicular and xylary fibre-specific expression in inflorescence stems, while reporter constructs of the woody plant-derived promoters were partial to the (pro)cambium-phloem and protoxylem. In transgenic *P. tremula* × *P. alba* plants, all three orthologous *SND1* promoters expressed the GUS reporter similarly and preferentially in developing secondary xylem, ray parenchyma and cork cambium. Ours is the first study to reciprocally test orthologous *SND1* promoter specificity in herbaceous and woody species, revealing diverged regulatory functions in the herbaceous system.

## 1. Introduction

The bioengineering of secondary cell walls (SCWs) in woody plants is a highly active area of research that relies on a thorough understanding of the complexity of SCW transcriptional regulation. The cellulose, hemicellulose and lignin biopolymers in secondary xylem (SX), which represent the bulk of plant biomass, are intrinsic to the production of feedstocks for bioeconomy products such as pulp, paper, biomaterials and second-generation biofuels [1,2,3] in addition to significantly influencing atmospheric carbon dioxide levels [4,5]. The ability to precisely modify wood development is, however, hampered by limited knowledge of the regulatory mechanisms involved [6].

The biosynthesis of SCW biopolymers in woody plants is regulated by a semi-hierarchical transcriptional network [7,8]. Certain NAC transcription factors (TFs) in this network, collectively known as secondary wall NACs (SWNs) [9], have been classified as SCW master switches in *Arabidopsis thaliana* (Arabidopsis). One of them, secondary wall-associated NAC domain protein 1 (SND1), also known as NAC secondary wall thickening promoting factor 3 (NST3) or ANAC012, yielded a predominantly interfascicular and xylary fibre-specific SCW reduction phenotype following dual knock-out of *SND1* and its functionally redundant homolog *NST1* [10,11]. This led to an interpretation of a fibre-specific role for SND1 in SCW regulation, although silique valve and endothecium SCWs were also reportedly affected [12,13]. The activity of the *SND1* promoter appears specific to xylem, with fragments of the *SND1* promoter ranging from ~1.8 kb to ~3.0 kb found to confer strong expression in the (pro)cambium, interfascicular fibres, metaxylem (especially xylary fibres) and xylem parenchyma cells of mature Arabidopsis inflorescence and hypocotyl stems [10,14,15]. To date, we have a poor understanding of the transcription factors and associated *cis*-regulatory elements that regulate the *SND1* promoter; among the few reported interactions in Arabidopsis are those of LBD30 [16], transcriptional repressors MYB4, MYB7 and MYB32 [17] as well as SND1 itself [17].

SND1 homologs such as *Populus trichocarpa* wood associated NAC Domain protein 1A (PtrWND1A) and PtrWND1B have also been functionally characterized in the woody angiosperms *Populus* [18,19,20,21,22,23,24] and *Eucalyptus* (EgrNAC61) [25,26,27], monocots [28,29] as well as the gymnosperms *Pinus pinaster* [30] and *Picea glauca* [31]. In poplar, *SND1* homologs do not affect fibre development exclusively: while quadruple knock-out of *NST1*/*NST2*/*SND1* co-orthologs *PtrWND1A*, *PtrWND1B*, *PtrWND2A* and *PtrWND2B* resulted in a loss of SCW deposition in xylem fibres, phloem fibres and ray parenchyma without affecting vessel SCW deposition [24], dominant repression of *PtrWND1B* reduced both fibre and vessel SCW deposition [25] and knock-down of *PtrWND1B* alone resulted in a reduction in fibre SCW thickness but an increase in vessel SCW thickness in poplar [22]. At the transcript level, *Populus SND1* homologs are expressed to similar degrees in developing fibres, vessels and phloem fibres as determined by in situ hybridisation and laser capture microdissection [19,32,33]. High-resolution transcript profiles of aspen early phloem, cambium and xylem layers [34] showed that *SND* homologs peak in the early developing xylem [35]. These expression profiles seem to agree with *SND1* promoter activity in woody systems: when the Arabidopsis *SND1* promoter was introduced into *Populus*, it conferred β-glucuronidase (GUS) reporter expression in the developing xylem similar to that of its endogenous profile in Arabidopsis [36]. Hence, we can hypothesize that the *SND1* promoter and those of its orthologs are activated in a conserved developing xylem-preferential fashion across diverged dicot lineages, both woody and non-woody.

Effective biotechnology strategies to improve lignocellulosic biomass require the development of high-precision biological parts, many of which are now available to us in the form of functionally characterized TFs occupying the SCW transcriptional network [37]. Since controlled spatiotemporal regulation of transgene expression mitigates the problem of ectopic expression, which may result in undesirable traits or loss of plant viability [38,39], the functional characterisation of xylogenesis-related promoter sequences diversifies the molecular toolkit at our disposal for woody biomass engineering. The regulatory functions of various promoter regions of SCW-related *Cellulose Synthase*, GT43 family, monolignol, and TF genes have been described to date, for example, [10,14,35,36,40,41,42,43,44,45,46,47,48]. However, a sound knowledge of the regulation of SCW-related promoters in different heterologous backgrounds, as well as the regulatory conservation of orthologous gene promoters from diverged angiosperm lineages is required to assess their potential use as standard biological parts with a universal syntax [49] in plant biotechnology.

In this study, we aimed to investigate the conservation (that is, the similarity and consistency) of spatial expression patterns of orthologous *SND1* promoter sequences from Arabidopsis (*AthSND1pro*), *Populus alba × P. grandidentata* (*PagWND1Apro*) and *Eucalyptus grandis* (*EgrNAC61pro*) in herbaceous and woody backgrounds. We show that, based on GUS reporter analysis, gene constructs containing the promoter regions of *AthSND1*, *EgrNAC61* and *PagWND1A* are similarly expressed in the developing xylem and cork cambium regions of transgenic *P. tremula* x *P. alba* plants, while the woody plant promoters lack xylem-specific expression in Arabidopsis and are hence regulated by a different mechanism. We also describe the expression patterns of Arabidopsis *CesA4* and *CesA7* gene promoters in hybrid poplar.

## 2. Results

### 2.1. Phylogenetic Analysis of SWN Proteins from Arabidopsis, Eucalyptus and Populus

**Figure 1:**
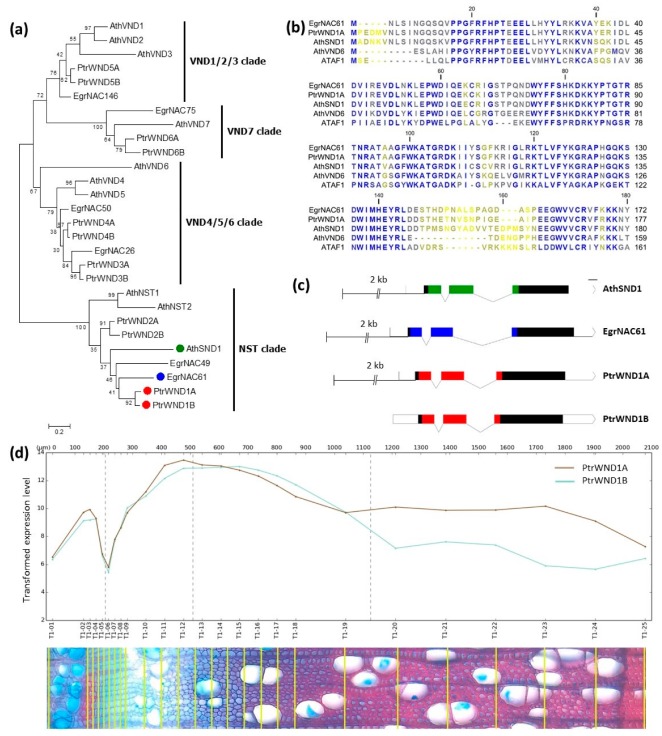
Phylogenetic and sequence analysis of NST and VND proteins. (**a**) Maximum likelihood phylogenetic tree. The tree with the highest log likelihood (−7877.59) is shown, drawn to scale, with branch lengths representing the number of substitutions per site. (**b**) Sequence alignment of the NAC domain of EgrNAC61, PtrWND1A and AthSND1. AthVND6 is included as a non-orthologous SWN, while ATAF1 is an outgroup NAC protein not known to regulate secondary cell walls (SCW) formation. Highly conserved residues are indicated in blue, semi-conserved residues in grey and variable residues in yellow. (**c**) Schematic representation of exon-intron structures and targeted promoter regions (where applicable) of the orthologous *SND1* promoters in this study. Coloured regions indicate the NAC domain while solid black shading indicates the C-terminal protein region; unshaded regions indicate untranslated regions. Scale bar = 10 amino acids. *Eucalyptus grandis* and *Populus trichocarpa* sequences are indicated with the prefixes Ath, Egr or Ptr, and green, blue and red shading, respectively. (**d**) AspWood (adapted from [34] with permission) transcript abundance profile of *PtrWND1A* and *PtrWND1B*. Micrograph measurements (in µm) are given on the upper edge of the graph.

We analysed the evolutionary relationships between orthologous and paralogous SWN proteins through a maximum likelihood approach, using the full-length protein sequences of group Ic NAC proteins excluding the SOMBRERO/BEARSKIN (SMB/BRN) clade [50]. This approach differs from a previous family-wide analysis of Arabidopsis, *Eucalyptus grandis* and *Populus trichocarpa* homologs based only on the conserved NAC domain [26]. We obtained a similar tree topology between Arabidopsis and *Populus* WND1 proteins according to previous reports [19,32], and a topology congruent with that of our previous family-wide NAC protein analysis [26], which included the *Eucalyptus* SWN proteins (Figure 1a). While a well-supported NST clade was evident, recent independent whole-genome duplication events in Arabidopsis [51], *Eucalyptus* [52] and *Populus* [53] challenged the identification of individual orthologous pairs. Our analysis identified EgrNAC49 and EgrNAC61 as co-orthologs of AthSND1, AthNST1 and AthNST2 as well as PtrWND1A/B and PtrWND2A/B. However, in light of the somewhat higher resolution obtained by Hussey et al. [26] which exclusively assigned EgrNAC49 as an AthNST1 ortholog, we regarded EgrNAC61, AthSND1 and PtrWND1A/B as co-orthologs in this study. This designation is further corroborated by their membership of an orthologous group according to the OrthoMCL [54] analysis method in PLAZA3.0 [55].

Alignment of the conserved NAC domain between EgrNAC61, PtrWND1A, AthSND1, a vessel-specific subfamily Ic outgroup AthVND6 and a distantly related NAC protein ATAF1 showed a more similar amino acid sequence for the orthologous proteins (Figure 1b). Furthermore, visualization of gene structures of EgrNAC61, AthSND1 and the in-paralogs PtrWND1A/B indicated a highly conserved exon-intron arrangement and DNA binding NAC domain lengths, with more variable second intron and C-terminal domain lengths (Figure 1c). For reporter gene analysis we delineated ~2 kb of promoter sequence for each ortholog, taken from the translation initiation codon, which included a similar length (~200 bp) of predicted 5′ UTR sequence. The *AthSND1* cloned promoter length was thus in between that of Ko et al. (~1.8 kb) [15] and Mitsuda et al. (~3.0 kb) [10]. The cloned *EgrNAC61pro* sequence was ~96% identical to the *E. grandis* BRASUZ1 reference [52], with two deletions and two insertions in the amplified allele (Figure S1). We only considered the promoter sequence of PtrWND1A as the *Populus* SND1 co-ortholog in this study, since the expression profiles of *PtrWND1A* and *PtrWND1B* across secondary vascular tissues of aspen wood were similar and both reached their peak during early SCW deposition according to the AspWood resource [34] (Figure 1d), suggesting their promoters are functionally redundant. We amplified an orthologous *PtrWND1A* sequence in a widely adopted hybrid, *P. alba* × *P. grandidentata* clone P39, herein named *PagWND1A*. The cloned sequence was evidently the *P. grandidentata* allele, with >99% identity to the reference *P. grandidentata* sequence (Figure S2).

### 2.2. Tissue-Specific Expression of Orthologous SND1 Promoters in Arabidopsis Inflorescence Stems and Hypocotyls

**Figure 2:**
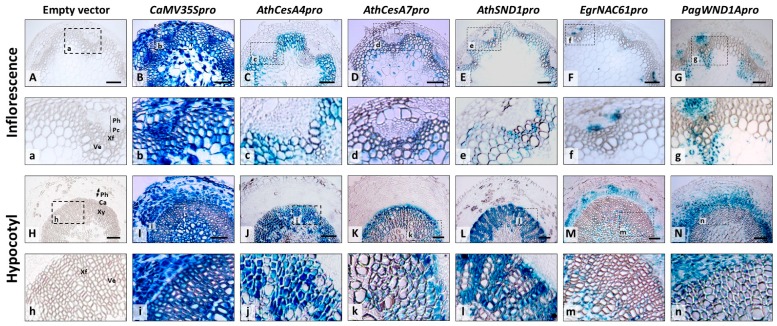
Tissue-specific expression analysis of *SND1* orthologous promoters in Arabidopsis inflorescence and hypocotyl stems. Representative GUS reporter analyses of six week-old Arabidopsis plants are shown. Panels (**A**–**G**) indicate inflorescence stem cross sections, where panels (**a**–**g**) represent magnified portions of the regions indicated by dashed-line boxes in panels (**A**–**G**), respectively. Panels (**H**–**N**) indicate hypocotyl stem cross sections, with panels (**h**–**n**) representing magnified version of the dashed-line boxes in panels (**H**–**N**), respectively. Ca, cambium; Pc, procambium; Ph, phloem; Ve, vessel; Xf, xylary fibre; Xy, xylem. Scale bar = 100 µm.

The tissue-specific expression of *AthSND1pro*, *EgrNAC61pro* and *PagWND1Apro* constructs were further studied in non-elongating stems of Arabidopsis plants that were approximately halfway through the reproductive phase. We report a summary of the results of the *promoter::reporter* analyses for all transgenic Arabidopsis lines in Table 1. The constitutive control promoter (*CaMV35S*) conferred e*GFP-GUS* expression in all tissues, while the empty vector control produced no GUS signal (Figure 2). The *AthCesA4pro* and *AthCesA7pro* markers, in contrast, directed expression to the xylem of the inflorescence stem and hypocotyl, with *AthCesA4pro* expression being more pronounced in the mature hypocotyl xylem, and *AthCesA7pro* partial to the developing SX of the hypocotyl (Figure 2j,k). The ~2 kb *AthSND1pro* fragment directed GUS activity in a similar way to that reported by Zhong et al. [14] and Mitsuda et al. [10] where reporter activity was pronounced in xylem, especially the fibre cells, of non-elongating inflorescence stems and hypocotyls (Figure 2e,l; Table 1).

It was previously reported that *EgrNAC61pro* was active in the vascular cylinder of *Arabidopsis* seedling hypocotyls, including proto- and metaxylem vessels [27]. Interestingly, however, in mature plants the woody plant promoters (*EgrNAC61pro* and *PagWND1Apro*) directed GUS expression preferentially in the procambium, phloem and protoxylem of non-elongating inflorescence stems (Figure 2f,g), as well as the cambium-phloem-cortex region of mature hypocotyls (Figure 2m,n; Table 1). Expression in fibres was observed only in some instances, mostly in the hypocotyl (Table 1), although *PagWND1Apro* directed expression in vascular bundle fibres as well (Figure 2g). These results indicate that the *SND1* orthologous promoters from the woody plant species do not confer the comparatively fibre-preferential specificity of the *AthSND1* promoter in Arabidopsis.

### 2.3. Analysis of Orthologous SND1 Promoters in Hybrid Poplar Trees

**Figure 3:**
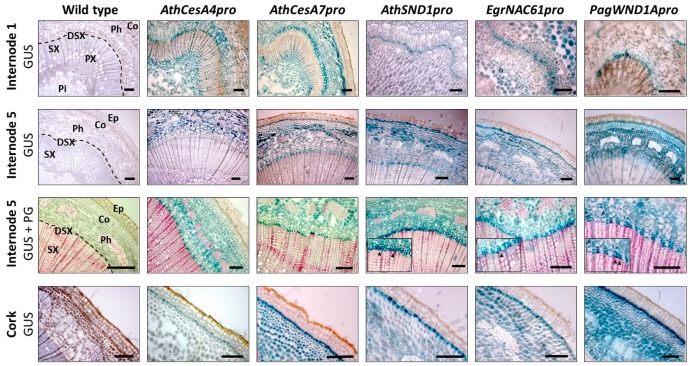
Histochemical analysis of transgenic *Populus tremula* × *P. alba* stems**.** Blue staining indicates β-glucuronidase (GUS) expression patterns, while phloroglucinol (PG) staining marks lignified cells in pink. The dashed line marks the approximate position of the vascular cambium. Inset images show higher magnification of GUS expression in developing xylem, where arrows indicate GUS expression in developing vessels. Ep, epidermis; Co, cortex; DSX, developing secondary xylem; Ph, phloem; Pi, pith; PX, primary xylem; SX, secondary xylem. Scale bar = 100 µm.

The promoter activities of *AthSND1pro*, *EgrNAC61pro* and *PagWND1Apro* were next assessed in a woody model, hybrid poplar (*Populus tremula* × *P. alba*) alongside *AthCesA4pro* and *AthCesA7pro* as early SCW deposition markers. At the time of sampling (late summer), the vascular cambium was not active and the cambial layer was not pronounced in the micrographs. However, phloroglucinol staining clearly distinguished lignified SX as well as phloem fibres (Figure 3, panel 3), allowing us to infer the position of the developing SX region. *AthCesA4pro* and *AthCesA8pro* transgenic trees showed GUS expression predominantly in developing SX, ray parenchyma and primary xylem of the first internode (Figure 3, panel 1). Compared to the *CesA* promoters, all the orthologous *SND1* promoter constructs showed more specific expression in the developing SX region of internode 1 (Figure 3), closely matching the *AthSND1pro* expression profile reported by Takata et al. [36]. We did not observe orthologous *SND1* promoter activity in the phloem fibres.

In internode 5, where SX deposition has been well established, *AthCesA4* and *AthCesA7* promoters specified GUS expression mostly in the developing SX region, with detectable expression in the cortex and strong expression in the cork cambium which gives rise to the phellem (Figure 3 panels 2–4). The orthologous *SND1* promoters conferred GUS expression similarly to the *AthCesA* promoters, where GUS expression was particularly pronounced in the cambium-developing SX region as well as the cork cambium (Figure 3, panels 2–4). Developing SX-specific expression appeared to be present in both developing fibres and vessels, and no GUS expression was observed in the phloem fibres for either the *CesA* or the orthologous *SND1* promoters. These data indicate that promoters of *cellulose synthase A* and orthologous *SND1* were more specific for developing xylem and cork development in the poplar background than in Arabidopsi.

## 3. Discussion

The functional diversity of SWN master regulators, and particularly their promoters, across angiosperm lineages is not well understood. While it has been demonstrated that SND1 orthologs from woody and herbaceous angiosperms, monocots and even gymnosperms perform similar roles in regulating SCW deposition [18,19,20,21,22,23,28,30,31,56], the transcriptional control of *SND1* expression and how those of its orthologs differ between plant lineages is less well characterised. The prospect of developing fibre-specific promoters as standardized biological parts for synthetic biology applications ideally requires that such promoters are universally modular and confer similar expression patterns in different heterologous hosts. However, gene duplication events and whole genome duplications in particular, can enable transcriptional network re-wiring and evolution [57]. This is seen, for example, in the diverged transcriptional activation of duplicated SCW *CesA* genes in *Populus* [58]. SCW-related promoters may in some instances behave unpredictably in a heterologous background where independent SCW transcriptional network re-wiring has occurred, impeding the translation of well-studied model promoter sequences to non-model crops. In this study, we aimed to characterize the spatial expression patterns of *SND1* orthologous promoters from woody and herbaceous plants in Arabidopsis and *Populus* backgrounds. One strength of our experimental approach is the reciprocal analysis of the same promoter constructs in an herbaceous and a woody host. In this way, at least one promoter’s expression is cisgenic in each background to serve as an endogenous reference, in addition to the inclusion of SCW *cellulose synthase* control promoters.

In one of the first analyses of the *SND1* promoter in Arabidopsis, the promoter was active in interfascicular fibres followed by metaxylem [14]. It was later found that the *SND1* promoter is also active in the vasculature and shoot of the seedling, as well as the procambium, xylem parenchyma, leaf veins, silique base and anther endothecium [10,12,15]. There are conflicting reports of whether *SND1* expression in xylem is fibre-specific in Arabidopsis, however, with some claims of expression in both fibres and vessels [10], and others claiming fibre-specific expression, at least in non-elongating inflorescence stem internodes [14]. Our *AthSND1pro* results closely matched the fibre-specific result of the latter study in non-elongating stems and hypocotyls (Figure 2e,l). This was despite a considerably shorter ~2 kb promoter used in our study compared to that of Mitsuda et al. [10] and Zhong et al. [14]. In poplar stems, we were surprised not to observe any *SND1* orthologous promoter activity in phloem fibres (Figure 3) given that the *AthSND1* promoter is active in these cells in poplar [36]. This may be explained by the possible lack of phloem fibre-specific elements in our ~2 kb promoter fragment. Another explanation is the fact that the trees were sampled in late summer following the cessation of phloem fibre development. It is known that phloem fibres begin and cease differentiating before that of xylem in *P. tremuloides* [59]. In support of the latter explanation, we did not observe evidence of SCW-associated *CesA* promoter expression in phloem fibres (Figure 3), suggesting that SCW deposition had ceased.

We first reported cork cambium expression of the *EgrNAC61* promoter in poplar stems [27]. In this study we observed that the *AthSND1* promoter, is also active in poplar cork cambium (Figure 3). Under appropriate short-day conditions, the Arabidopsis hypocotyl also develops a cork cambium (phellogen) [60], although *AthSND1pro* activity has not yet been studied under such conditions. Nonetheless, the similar preferential expression in poplar cork cambial tissue of *AthSND1pro* compared to the woody plant *SND1* orthologous promoters suggests a similar cis-regulatory mechanism. Cork (phellem) undergoes programmed cell death and has G-rich lignified cell walls in addition to suberin [61,62], and accordingly, the phenylpropanoid pathway and cell wall-associated transcripts are enriched among genes upregulated in poplar phellem [63]. Since SND1 regulates programmed cell death and lignin biosynthesis [14,48], we postulate that SND1 and its orthologs were co-opted, along with the transcriptional mechanisms activating their promoter, to activate these biological processes in developing cork.

The evidence gathered in this study demonstrates that promoters of *SND1* orthologs from Arabidopsis, *Eucalyptus* and *Populus* confer similar expression profiles preferentially in the developing xylem and cork cambium of poplar stems, while *SND1* promoters from *Eucalyptus* and *Populus* confer (pro)cambium-phloem-cortex and protoxylem-preferential expression in Arabidopsis relative to a largely fibre-specific expression pattern for *AthSND1pro*. Clearly, the orthologous *SND1* promoters showed highly similar expression consistent with a SCW regulatory function in poplar (Figure 3), but not the vascular tissue of non-elongating Arabidopsis stems (Figure 2). This disproves the hypothesis that the *SND1* promoter is similarly activated in all angiosperm lineages. We cannot currently explain why *EgrNAC61pro* and *PagWND1Apro* apparently lost fibre-specific expression and acquired procambium-phloem and protoxylem-preferential expression in Arabidopsis inflorescence stems. We postulate that Arabidopsis-specific transcriptional interactions achieve fibre-specific expression of *SND1* in Arabidopsis. However, our result is consistent with a recent report that a 315-bp *PtrWND1A* promoter drove GUS expression mostly in the procambium (and possibly the phloem) as well as the protoxylem of inflorescence stem vascular bundles in Arabidopsis, while a 1276-bp promoter fragment was entirely specific for the procambium-phloem [35]. Gibberellic acid application resulted in GUS expression extending into the phloem of Arabidopsis vascular bundles [35], indicating that phytohormones affect the promoter activity and specificity. We cannot rule out the possibility that in the current study and that of Johnsson et al. [35], the promoter sequences lack crucial upstream *cis*-elements required for fibre-specific expression in Arabidopsis. Nonetheless, we have shown that (1) *SND1* orthologous promoters in this study are all suitable candidates for achieving developing xylem and cork cambium-specific expression in poplar, (2) that *AthSND1pro* has conserved activity in cork cambium with *EgrNAC61pro* and *PagWND1Apro*, and that (3) the *SND1* promoters from herbaceous and woody plants display divergent functions in different plant backgrounds, particularly in Arabidopsis.

## 4. Materials and Methods

### 4.1. Phylogenetic Analysis

NST and VND clade members from Arabidopsis, *Eucalyptus grandis* and *Populus trichocarpa* were aligned with MUSCLE [64] in MEGA6 [65] using default parameters. The alignment was subjected to phylogenetic analysis using the maximum likelihood method based on the JTT matrix-based model [66] and 1000 bootstrap iterations. A discrete Gamma distribution was used to model evolutionary rate differences among sites (5 categories). All positions with less than 95% site coverage were eliminated. For gene structure visualizations, predicted annotated features from Phytozome [67] and the Arabidopsis Information Resource TAIR10; [68] were submitted to http://wormweb.org/exonintron.

### 4.2. Reporter Construct Preparation

*Arabidopsis thaliana* ecotype Columbia (Col-0), *Eucalyptus grandis* W. Hill ex Maiden (Mondi clone TAG0014) and *Populus alba* x *P. grandidentata* clone P39 were used as genetic source materials for this study. Preparation of the *EgrNAC61pro* construct is described in [27]. Approx. 2 kb of sequence upstream of the start codon of *Arabidopsis thaliana SND1* (AthSND1; AT1G32770) and *Populus trichocarpa* (PtrWND1A; Potri.011G153300) genes were retrieved from the Phytozome database (www.phytozome.org) and used to design primers. Restriction sites KpnI and HindIII were added at the 5’ ends of each forward and reverse oligonucleotide, respectively. Regions amplified from genomic DNA using the Phusion high fidelity DNA polymerase (New England Biolabs, Ipswich, MA, USA) included 2009 bp spanning from -2000 to +9 relative to the translational initiation position for Arabidopsis (*AthSND1pro;* GenBank accession MH394193) and 2058 bp spanning from −2049 to +9 for *Populus alba* × *grandidentata* clone P39 (*PagWND1Apro;* GenBank accession MH394191) promoters. The PCR products were adenylated using *Taq* DNA polymerase (Roche CustomBiotech, Indianapolis, IN, ISA) and cloned into pCR8/GW/TOPO as per the manufacturer’s instructions (Invitrogen, Carlsbad, CA, USA). For Arabidopsis experiments, the inserts were transferred to the pBGWFS7 vector bearing the *eGFP:GUS* reporter gene [69] using the Gateway LR Clonase II Enzyme mix (Invitrogen). pCAMBIA2301 was used as positive control for constitutive GUS expression, while an empty pBGWFS7 vector was used as negative control. For transgenic poplar experiments, the inserts were transferred to pMDC162 [70]. The Arabidopsis CesA4 promoter (AT5G44030; GenBank accession MH394194) and *CesA7* promoter (*AthCesA7pro*) [43] were similarly cloned into pBGWFS7 and pMDC162 as markers for SCW deposition.

### 4.3. Plant Transformation and Growth

After verification by sequencing, the constructs were used to transform *Agrobacterium tumefaciens* strain AGL1. Arabidopsis ecotype Col-0 plants were transformed by the floral dip method [71]. The first generation of transgenic seeds was selected on soil mix by spraying the leaves of 10 days old seedlings with Basta (Glufosinate-ammonium PESTANAL, Sigma-Aldrich Corporation, St. Louis, MO, USA) at 100 mg L^–1^ and repeating every 3 days, 5 times. Homozygous lines were further selected on half-strength MS agar plates using Basta at 20 mg L^−1^. Positive control lines containing pCAMBIA2301 were similarly selected on 100 mg L^−1^ kanamycin. The putative transgenic plants were screened by genomic PCR to verify the presence of the constructs. Ten week-old transgenic Arabidopsis plants (3–6 lines of each construct) grown under long-day (16 h) photoperiod at 23 ± 1 °C, fluorescent white light intensity of 70–120 mM m^−2^ s^−1^ were sampled for GUS staining. This time period corresponded to the developmental stage midway between bolting and silique maturation. For poplar transformations (*P. alba* × *P. tremula* clone 717-1B4), we followed the leaf disk inoculation method described by Coleman et al. [72], with selection on hygromycin (30 mg L^−1^). Plants were grown and maintained in a greenhouse under long-day conditions (16 h light/8 h dark at 23 ± 1 °C) for approximately 3 months. Internodes 1 and 5 were harvested from transgenic trees in late summer for GUS reporter staining.

### 4.4. Microscopy and Histochemical Analysis

GUS staining was performed as previously reported [73], with modifications. For Arabidopsis experiments, 2–4 mm sections of inflorescence stem and hypocotyl were immersed in GUS staining buffer containing 100 mM sodium phosphate buffer (NaH_2_PO_4_ and Na_2_HPO_4_, pH 7.0), 0.5 mM potassium ferrocyanide, 0.5 mM potassium ferricyanide, 10 mM EDTA, 0.5% (*v*/*v*) Triton X-100 and 0.5 mM 5-bromo-4-chloro-3-indolyl-β-glucuronic acid (X-Gluc). For the *Populus* material, the modified protocol described by Spokevicius et al. [74] was adopted. The samples were incubated for 16 h at 37 °C in the dark. The staining buffer was removed, the samples were de-stained with an ethanol wash series over several days, and stored in 70% ethanol. The stained Arabidopsis seedlings, mature organs and stem sections were observed using Zeiss Lumar V12 dissection microscope (Carl Zeiss AG, Oberkochen, Germany). For inflorescence and hypocotyl stem material, the stained sections were dehydrated by a successive gradient of 90% ethanol, 100% ethanol (×2), ethanol/xylene (1:1 *v*/*v*) and 100% xylene (×2) for 1 h each. The dehydrated samples were transferred into a solution of paraffin wax/xylene (1:1 *v*/*v*) and incubated at 60 °C until xylene was completely evaporated, followed by transfer into molten 100% paraffin wax and casting into pyramid-shape wells. Sections of 8–20 µm were cut with a microtome and observed using a Zeiss Axio Imager M2 microscope (Carl Zeiss AG, Oberkochen, Germany). Stained *Populus* material was re-hydrated in sterile water for 24 h prior to imaging, sections were cut on a cryomicrotome, and subjected to bright field microscopy. Phloroglucinol-HCl histochemical staining was performed according to Mitra and Loqué [75]. ImageJ 1.48v (http://imagej.nih.gov/ij) and Helicon Focus (http://www.heliconsoft.com) software were used for image processing and focus stacking.

## Figures and Tables

**Table 1:** Summary of GUS reporter staining in transgenic Arabidopsis plants.

Constructs	Inflorescence Stem									Hypocotyl					
	Ep	Co	IF	Pc-Ph	Mx		Px		Pi	Ep	Co	Ca-Ph	Xy		Pi
					Xf	Ve	Xf	Ve					Xf	Ve	
*AthCesA4pro::eGFP-GUS* (*n* = 4)	-	-	+++	+	++	++	++	-	+	-	-	-	+++	++	-
*AthCesA7pro::eGFP-GUS* (*n* = 4)	-	+	+++	+	+++	++	++	-	+	-	-	-	+++	++	-
*AthSND1pro::eGFP-GUS* (*n* = 6)	-	-	+++	-	+++	-	+	-	-	-	-	-	+++	-	-
*EgrNAC61pro::eGFP-GUS* (*n* = 5)	-	-	-	+++	+	-	+++	-	-	-	+	+++	+++	-	+++
*PagWND1Apro::eGFP-GUS* (*n* = 4)	-	+	+	+++	+++	+	+++	+	+	-	+++	+++	+++	+	+++
*CaMV35S::GUS* (*n* = 3)	+++	+++	+++	+++	+++	+++	+++	+++	+++	+++	+++	+++	+++	+++	+++

GUS expression, assessed histologically, is summarized as: +++, expression detected in >75% of plants; ++, expression in 51–75% of plants; +, expression in 25–50% of plants; -, expression in 0% of plants. Ca-Ph, cambium-phloem; Co, cortex; Ep, epidermis; IF, interfascicular fibres; Mx, metaxylem; n, number of transgenic lines analysed; Pc-Ph, procambium-phloem; Pi, pith; Px, protoxylem; Ve, vessel; Xf, xylary fibre; Xy, xylem.

## Supplementary Materials

Supplementary materials can be found at https://www.mdpi.com/1422-0067/20/18/4623/s1.

## Abbreviations

*Ath*	*Arabidopsis thaliana*
BRN	bearskin
Ca	cambium
Co	cortex
DSX	developing secondary xylem
eGFP	enhanced Green Fluorescent Protein
*Egr*	*Eucalyptus grandis*
Ep	epidermis
GUS	β-glucuronidase
IF	interfascicular fibres
Mx	metaxylem
NST	NAC secondary wall thickening promoting factor
*Pag*	*Populus alba × P. grandidentata*
Pc	procambium
Ph	phloem
Pi	pith
*Ptr*	*Populus trichocarpa*
Px	protoxylem
PX	primary xylem
SCW	secondary cell wall
SND	secondary wall-associated NAC domain protein
SMB	somrero
SWN	Secondary Wall NAC
SX	secondary xylem
TF	transcription factor
UTR	untranslated region
Ve	vessels
VND	vascular related NAC domain
WND	wood associated NAC domain
Xf	xylary fibres
Xy	xylem

## References

[1] Carroll A., Somerville C. (2009). Cellulosic biofuels. Annu. Rev. Plant Biol..

[2] Loqué D., Scheller H.V., Pauly M. (2015). Engineering of plant cell walls for enhanced biofuel production. Curr. Opin. Plant Biol..

[3] Ragauskas A.J., Beckham G.T., Biddy M.J., Chandra R., Chen F., Davis M.F., Davison B.H., Dixon R.A., Gilna P., Keller M. (2014). Lignin valorization: Improving lignin processing in the biorefinery. Science.

[4] Thornley J.H.M., Cannell M.G.R. (2000). Managing forests for wood yield and carbon storage: A theoretical study. Tree Physiol..

[5] Lucas W.J., Groover A., Lichtenberger R., Furuta K., Yadav S.-R., Helariutta Y., He X.-Q., Fukuda H., Kang J., Brady S.M. (2013). The plant vascular system: Evolution, development and functions. J. Integr. Plant Biol..

[6] Didi V., Jackson P., Hejatko J. (2015). Hormonal regulation of secondary cell wall formation. J. Exp. Bot..

[7] Hussey S.G., Mizrachi E., Creux N.M., Myburg A.A. (2013). Navigating the transcriptional roadmap regulating plant secondary cell wall deposition. Front. Plant Sci..

[8] Schuetz M., Smith R., Ellis B. (2013). Xylem tissue specification, patterning, and differentiation mechanisms. J. Exp. Bot..

[9] Zhong R., Lee C., Ye Z.-H. (2010). Global analysis of direct targets of secondary wall NAC master switches in *Arabidopsis*. Mol. Plant.

[10] Mitsuda N., Iwase A., Yamamoto H., Yoshida M., Seki M., Shinozaki K., Ohme-Takagi M. (2007). NAC transcription factors, NST1 and NST3, are key regulators of the formation of secondary walls in woody tissues of *Arabidopsis*. Plant Cell.

[11] Zhong R., Richardson E.A., Ye Z.-H. (2007). Two NAC domain transcription factors, SND1 and NST1, function redundantly in regulation of secondary wall synthesis in fibers of *Arabidopsis*. Planta.

[12] Mitsuda N., Ohme-Takagi M. (2008). NAC transcription factors NST1 and NST3 regulate pod shattering in a partially redundant manner by promoting secondary wall formation after the establishment of tissue identity. Plant J..

[13] Mitsuda N., Seki M., Shinozaki K., Ohme-Takagi M. (2005). The NAC transcription factors NST1 and NST2 of Arabidopsis regulate secondary wall thickenings and are required for anther dehiscence. Plant Cell.

[14] Zhong R., Demura T., Ye Z.H. (2006). SND1, a NAC domain transcription factor, is a key regulator of secondary wall synthesis in fibers of *Arabidopsis*. Plant Cell.

[15] Ko J.-H., Yang S.H., Park A.H., Lerouxel O., Han K.-H. (2007). ANAC012, a member of the plant-specific NAC transcription factor family, negatively regulates xylary fiber development in *Arabidopsis thaliana*. Plant J..

[16] Liu C., Yu H., Li L. (2019). SUMO modification of LBD30 by SIZ1 regulates secondary cell wall formation in *Arabidopsis thaliana*. PLoS Genet..

[17] Wang H., Zhao Q., Chen F., Wang M., Dixon R.A. (2011). NAC domain function and transcriptional control of a secondary cell wall master switch. Plant J..

[18] Zhong R., Ye Z.H. (2010). The poplar PtrWNDs are transcriptional activators of secondary cell wall biosynthesis. Plant Signal. Behav..

[19] Zhong R., Lee C., Ye Z.H. (2010). Functional characterization of poplar wood-associated NAC domain transcription factors. Plant Physiol..

[20] Li Q., Lin Y.C., Sun Y.H., Song J., Chen H., Zhang X.H., Sederoff R.R., Chiang V.L. (2012). Splice variant of the SND1 transcription factor is a dominant negative of SND1 members and their regulation in *Populus trichocarpa*. Proc. Natl. Acad. Sci. USA.

[21] Lin Y.-C., Li W., Sun Y.-H., Kumari S., Wei H., Li Q., Tunlaya-Anukit S., Sederoff R.R., Chiang V.L. (2013). SND1 transcription factor—Directed quantitative functional hierarchical genetic regulatory network in wood formation in *Populus trichocarpa*. Plant Cell.

[22] Zhao Y., Sun J., Xu P., Zhang R., Li L. (2014). Intron-mediated alternative splicing of WOOD-ASSOCIATED NAC TRANSCRIPTION FACTOR1B regulates cell wall thickening during fiber development in *Populus* species. Plant Physiol..

[23] Yang L., Hou Y., Zhao X., Lu W., Li Y., Yang F., Tang S., Luo K. (2015). Identification and characterization of a wood-associated NAC domain transcription factor PtoVNS11 from Populus tomentosa Carr. Trees.

[24] Takata N., Awano T., Nakata M.T., Sano Y., Sakamoto S., Mitsuda N., Taniguchi T. (2019). Populus NST/SND orthologs are key regulators of secondary cell wall formation in wood fibers, phloem fibers and xylem ray parenchyma cells. Tree Physiol..

[25] Zhong R., McCarthy R.L., Lee C., Ye Z.-H. (2011). Dissection of the transcriptional program regulating secondary wall biosynthesis during wood formation in poplar. Plant Physiol..

[26] Hussey S.G., Saidi M.N., Hefer C.A., Myburg A.A., Grima-Pettenati J. (2015). Structural, evolutionary and functional analysis of the NAC domain protein family in *Eucalyptus*. New Phytol..

[27] Laubscher M., Brown K., Tonfack L.B., Myburg A.A., Mizrachi E., Hussey S.G. (2018). Temporal analysis of *Arabidopsis* genes activated by *Eucalyptus grandis* NAC transcription factors associated with xylem fibre and vessel development. Sci. Rep..

[28] Golfier P., Volkert C., He F., Rausch T., Wolf S. (2017). Regulation of secondary cell wall biosynthesis by a NAC transcription factor from *Miscanthus*. Plant Direct.

[29] Zhong R., Lee C., McCarthy R.L., Reeves C.K., Jones E.G., Ye Z.-H. (2011). Transcriptional activation of secondary wall biosynthesis by rice and maize NAC and MYB transcription factors. Plant Cell Physiol..

[30] Pascual M.B., Llebrés M.T., Craven-Bartle B., Cañas R.A., Cánovas F.M., Ávila C. (2017). PpNAC1, a main regulator of phenylalanine biosynthesis and utilization in maritime pine. Plant Biotechnol. J..

[31] Duval I., Lachance D., Giguère I., Bomal C., Morency M.J., Pelletier G., Boyle B., MacKay J.J., Séguin A. (2014). Large-scale screening of transcription factor-promoter interactions in spruce reveals a transcriptional network involved in vascular development. J. Exp. Bot..

[32] Ohtani M., Nishikubo N., Xu B., Yamaguchi M., Mitsuda N., Goue N., Shi F., Ohme-Takagi M., Demura T. (2011). A NAC domain protein family contributing to the regulation of wood formation in poplar. Plant J..

[33] Lin Y.J., Chen H., Li Q., Li W., Wang J.P., Shi R., Tunlaya-Anukit S., Shuai P., Wang Z., Ma H. (2017). Reciprocal cross-regulation of VND and SND multigene TF families for wood formation in *Populus trichocarpa*. Proc. Natl. Acad. Sci. USA.

[34] Sundell D., Street N.R., Kumar M., Mellerowicz E.J., Kucukoglu M., Johnsson C., Kumar V., Mannapperuma C., Delhomme N., Nilsson O. (2017). AspWood: High-spatial-resolution transcriptome profiles reveal uncharacterized modularity of wood formation in *Populus tremula*. Plant Cell.

[35] Johnsson C., Jin X., Xue W., Dubreuil C., Lezhneva L., Fischer U. (2018). The plant hormone auxin directs timing of xylem development by inhibition of secondary cell wall deposition through repression of secondary wall NAC-domain transcription factors. Physiol. Plant..

[36] Takata N., Sakamoto S., Mitsuda N., Taniguchi T. (2017). The *Arabidopsis NST3/SND1* promoter is active in secondary woody tissue in poplar. J. Wood Sci..

[37] Kalluri U.C., Yin H., Yang X., Davison B.H. (2014). Systems and synthetic biology approaches to alter plant cell walls and reduce biomass recalcitrance. Plant Biotechnol. J..

[38] Moummou H., Tonfack L.B., Chervin C., Benichou M., Youmbi E., Ginies C., Latché A., Pech J.C., van der Rest B. (2012). Functional characterization of SlscADH1, a fruit-ripening-associated short-chain alcohol dehydrogenase of tomato. J. Plant. Physiol..

[39] Etchells J.P., Mishra L.S., Kumar M., Campbell L., Turner S.R. (2015). Wood Formation in Trees Is Increased by Manipulating PXY-Regulated Cell Division. Curr. Biol..

[40] Ratke C., Pawar P.M.-A., Balasubramanian V.K., Naumann M., Duncranz M.L., Derba-Maceluch M., Gorzsás A., Endo S., Ezcurra I., Mellerowicz E.J. (2015). *Populus GT43* family members group into distinct sets required for primary and secondary wall xylan biosynthesis and include useful promoters for wood modification. Plant Biotechnol. J..

[41] Xie L., Yang C., Wang X. (2011). Brassinosteroids can regulate cellulose biosynthesis by controlling the expression of *CESA* genes in *Arabidopsis*. J. Exp. Bot..

[42] Hauffe K.D., Paszkowski U., Schulze-Lefert P., Hahlbrock K., Dangl J.L., Douglas C.J. (1991). A parsley 4CL-1 promoter fragment specifies complex expression patterns in transgenic tobacco. Plant Cell.

[43] Smith R.A., Schuetz M., Roach M., Mansfield S.D., Ellis B., Samuels L. (2013). Neighboring parenchyma cells contribute to *Arabidopsis* xylem lignification, while lignification of interfascicular fibers is cell autonomous. Plant Cell.

[44] Li E., Bhargava A., Qiang W., Friedmann M.C., Forneris N., Savidge R.A., Johnson L.A., Mansfield S.D., Ellis B.E., Douglas C.J. (2012). The Class II KNOX gene KNAT7 negatively regulates secondary wall formation in *Arabidopsis* and is functionally conserved in *Populus*. New Phytol.

[45] Li E., Wang S., Liu Y., Chen J.-G., Douglas C.J. (2011). OVATE FAMILY PROTEIN4 (OFP4) interaction with KNAT7 regulates secondary cell wall formation in *Arabidopsis thaliana*. Plant J..

[46] Creux N.M., Ranik M., Berger D.K., Myburg A.A. (2008). Comparative analysis of orthologous cellulose synthase promoters from *Arabidopsis*, *Populus* and *Eucalyptus*: Evidence of conserved regulatory elements in angiosperms. New Phytol..

[47] Wang S., Li E., Porth I., Chen J.G., Mansfield S.D., Douglas C.J. (2014). Regulation of secondary cell wall biosynthesis by poplar R2R3 MYB transcription factor PtrMYB152 in *Arabidopsis*. Sci. Rep..

[48] Zhong R., Lee C., Zhou J., McCarthy R.L., Ye Z.-H. (2008). A battery of transcription factors involved in the regulation of secondary cell wall biosynthesis in *Arabidopsis*. Plant Cell.

[49] Patron N.J., Orzaez D., Marillonnet S., Warzecha H., Matthewman C., Youles M., Raitskin O., Leveau A., Farré G., Rogers C. (2015). Standards for plant synthetic biology: A common syntax for exchange of DNA parts. New Phytol.

[50] Zhu T., Nevo E., Sun D., Peng J. (2012). Phylogenetic analyses unravel the evolutionary history of NAC proteins in plants. Evolution.

[51] Van de Peer Y., Fawcett J.A., Proost S., Sterck L., Vandepoele K. (2009). The flowering world: A tale of duplications. Trends Plant Sci..

[52] Myburg A.A., Grattapaglia D., Tuskan G.A., Hellsten U., Hayes R.D., Grimwood J., Jenkins J., Lindquist E., Tice H., Bauer D. (2014). The genome of *Eucalyptus grandis*. Nature.

[53] Tuskan G.A., DiFazio S., Jansson S., Bohlmann J., Grigoriev I., Hellsten U., Putnam N., Ralph S., Rombauts S., Salamov A. (2006). The genome of black cottonwood, *Populus trichocarpa* (Torr. & Gray). Science.

[54] Li L., Stoeckert C.J., Roos D.S. (2003). OrthoMCL: Identification of ortholog groups for eukaryotic genomes. Genome Res..

[55] Proost S., Van Bel M., Vaneechoutte D., Van de Peer Y., Inze D., Mueller-Roeber B., Vandepoele K. (2015). PLAZA 3.0: An access point for plant comparative genomics. Nucleic Acids Res..

[56] Zhong R., Yuan Y., Spiekerman J.J., Guley J.T., Egbosiuba J.C., Ye Z.H. (2015). Functional Characterization of NAC and MYB Transcription Factors Involved in Regulation of Biomass Production in Switchgrass (*Panicum virgatum*). PLoS ONE.

[57] Voordeckers K., Pougach K., Verstrepen K.J. (2015). How do regulatory networks evolve and expand throughout evolution?. Curr. Opin. Biotechnol..

[58] Takata N., Taniguchi T. (2015). Expression divergence of cellulose synthase (*CesA*) genes after a recent whole genome duplication event in *Populus*. Planta.

[59] Davis J.D., Evert R.F. (1968). Seasonal Development of the Secondary Phloem in *Populus tremuloides*. Bot. Gaz..

[60] Chaffey N., Cholewa E., Regan S., Sundberg B. (2002). Secondary xylem development in *Arabidopsis*: A model for wood formation. Physiol. Plant..

[61] Marques A.V., Pereira H., Meier D., Faix O. (1999). Structural Characterization of Cork Lignin by Thioacidolysis and Permanganate Oxidation. Holzforschung.

[62] Jones R., Ougham H., Thomas H., Waaland S. (2013). The Molecular Life of Plants.

[63] Rains M.K., Gardiyehewa de Silva N.D., Molina I. (2018). Reconstructing the suberin pathway in poplar by chemical and transcriptomic analysis of bark tissues. Tree Physiol..

[64] Edgar R.C. (2004). MUSCLE: Multiple sequence alignment with high accuracy and high throughput. Nucleic Acids Res..

[65] Tamura K., Stecher G., Peterson D., Filipski A., Kumar S. (2013). MEGA6: Molecular Evolutionary Genetics Analysis version 6.0. Mol. Biol. Evol..

[66] Jones D.T., Taylor W.R., Thornton J.M. (1992). The rapid generation of mutation data matrices from protein sequences. Bioinformatics.

[67] Goodstein D.M., Shu S.Q., Howson R., Neupane R., Hayes R.D., Fazo J., Mitros T., Dirks W., Hellsten U., Putnam N. (2012). Phytozome: A comparative platform for green plant genomics. Nucleic Acids Res..

[68] Lamesch P., Berardini T.Z., Li D., Swarbreck D., Wilks C., Sasidharan R., Muller R., Dreher K., Alexander D.L., Garcia-Hernandez M. (2012). The Arabidopsis Information Resource (TAIR): Improved gene annotation and new tools. Nucleic Acids Res..

[69] Karimi M., Inzé D., Depicker A. (2002). GATEWAY™ vectors for *Agrobacterium*-mediated plant transformation. Trends in Plant Science.

[70] Curtis M.D., Grossniklaus U. (2003). A gateway cloning vector set for high-throughput functional analysis of genes in planta. Plant Physiol..

[71] Clough S.J., Bent A.F. (1998). Floral dip: A simplified method for *Agrobacterium*-mediated transformation of *Arabidopsis thaliana*. Plant J..

[72] Coleman H.D., Canam T., Kang K.Y., Ellis D.D., Mansfield S.D. (2007). Over-expression of UDP-glucose pyrophosphorylase in hybrid poplar affects carbon allocation. J. Exp. Bot..

[73] Jefferson R.A., Kavanagh T.A., Bevan M.W. (1987). GUS fusions: β-glucuronidase as a sensitive and versatile gene fusion marker in higher plants. EMBO J..

[74] Spokevicius A.V., van Beveren K.S., Bossinger G. (2006). *Agrobacterium*-mediated transformation of dormant lateral buds in poplar trees reveals developmental patterns in secondary stem tissues. Funct. Plant Biol..

[75] Pradhan Mitra P., Loqué D. (2014). Histochemical staining of *Arabidopsis thaliana* secondary cell wall elements. J. Vis. Exp..

