# Supplementary material for: Analysis of Orthologous SECONDARY WALL-ASSOCIATED NAC DOMAIN1 (SND1) Promotor Activity in Herbaceous and Woody Angiosperms
Source: Int J Mol Sci. 2019 Sep 18;20(18):4623. doi: 10.3390/ijms20184623 (PMC6770381; doi:10.3390/ijms20184623)
Supplement: Supplementary file 1 [file ijms-20-04623-s001.pdf]

Figure S1. Alignment of the cloned *EgrNAC61pro* sequence against the *E. grandis* v.2.0 clone BRASUZ1 sequence (Myburg *et al.* 2014).

```
                                                          20                            40                            60
EgrNAC61pro cloned     CATGTGTGCG TTTGAGAGAG AGAGAGAGAG AGAG------ ---------- --TAGGGGAC 42
EgrNAC61pro reference  CATGTGTGCG TTTGAGAGAG AGAGAGAGAG AGAGAGAGAG AGAGAGAGAG AGTAGGGGAC 60
                                                          80                           100                           120
EgrNAC61pro cloned     AGTGTGGTAG TTTCGGGGGT GCTTTTCATA TAGATTTTAA GGTCGAGGGT CATAGTTTTT 102
EgrNAC61pro reference  AGTGTGGTAG TTTCGGGGGT GCTTTTCATA TAGATTTTAA GGTCGAGGGT CATAGTTTTT 120
                                                         140                           160                           180
EgrNAC61pro cloned     CGATGGAGCC CTGCCCTCCT CTTTCGGCTA TGGTTATTTG TTGCTTCTTG TAACGCATTT 162
EgrNAC61pro reference  CGATGGAGCC CTGCCCTCCT CTTTCGGCTA TGGTTATTTG TTGCTTCTTG TAACGCATTT 180
                                                         200                           220                           240
EgrNAC61pro cloned     TTTGGGTAAT CCAGAAAGAT CGTGGGACCC CATCTTCCCC GGCCTCTCTG TTCAGGATTA 222
EgrNAC61pro reference  TTTGGGTAAT CCAGAAAGAT CGTGGGACCC CATCTTCCCC GGCCTCTCTG TTCAGGATTA 240
                                                         260                           280                           300
EgrNAC61pro cloned     CCATCATATA ATCACGTTTG CTACTGAGGA GGAAGCTGGG ATACACCAGA TAACCACGAG 282
EgrNAC61pro reference  CCATCATATA ATCACGTTTG CTACTGAGGA GGAAGCTGGG ATACACCAGA TAACCACGAG 300
                                                         320                           340                           360
EgrNAC61pro cloned     CAAGTTGATC CAGCGGGTTG ATGAATTCGC TTTTCTTTTG GAAAAGTTGT GGGGGCTTCA 342
EgrNAC61pro reference  CAAGTTGATC CAGCGGGTTG ATGAATTCGC TTTTCTTTTG GAAAAGTTGT GGGGGCTTCA 360
                                                         380                           400                           420
EgrNAC61pro cloned     GGTCGCTAAA TACAGCAGTA CCATATAAAC AGGTCCTCTC GATACGACCT TTTGTCCAAC 402
EgrNAC61pro reference  GGTCGCTAAA TACAGCAGTA CCATATAAAC AGGTCCTCTC GATACGACCT TTTGTCCAAC 420
                                                         440                           460                           480
EgrNAC61pro cloned     AGCGGATGGA AAATGCGGAG GAGCTGGAGA TCGACAGTGT ACGGATACTA TAGCAGATTC 462
EgrNAC61pro reference  AGCGGATGGA AAATGCGGAG GAGCTGGAGA TCGACAGTGT ACGGATACTA TAGCAGATTC 480
                                                         500                           520                           540
EgrNAC61pro cloned     GTCCTGCCTT TTGATTGCGA TACCCACTCG CTTACCTAGC ACACAAAAAA AAAAAAAAAA 522
EgrNAC61pro reference  GTCCTGCCTT TTGATTGCGA TACCCACTCG CTTACCTAGC ACACAAAAAA AAAAAAAAAA 540
                                                         560                           580                           600
EgrNAC61pro cloned     AAAAAAATTA GTTTTAAAAT TTTTTGGTGT TGATCAATAA CGGAAAAGAT TGGATTTTGT 582
EgrNAC61pro reference  AAA----TTA GTTTTAAAAT TTTTTGGTGT TGATCAATAA CGGAAAAGAT TGGATTTTGT 596
                                                         620                           640                           660
EgrNAC61pro cloned     GCGGCAATAA TATTTAGGTA GTGAATATGT TCGCTTATCG GTTTTCCATT TTAATCATTT 642
EgrNAC61pro reference  GCGGCAATAA TATTTAGGTA GTGAATATGT TCGCTTATCA GTTTTCCATT TTAATCATTT 656
                                                         680                           700                           720
EgrNAC61pro cloned     TACGTAATAA ATTGGTTGGC GATATAACTT TTATTTTGAT AGCGATAAAT CTCAATTAGG 702
EgrNAC61pro reference  TACGTAATAA ATTGGCTGGC GATATAACTT TTATTTTGAT AGCGATAAAT CTCAATTAGG 716
                                                         740                           760                           780
EgrNAC61pro cloned     TATATTTGAG TTTCAATTCT TTGAAGAGTT GAATATTTTT CATTACGGAA GGGTAAGGGT 762
EgrNAC61pro reference  TATATTTGAG TTTCAATTCT TTGAAGAGTT GAATATTTTT CATTACGGAA GGGTAAGGGT 776
                                                         800                           820                           840
EgrNAC61pro cloned     AAATTCCTTA TTTTTGTAAC TTACAAAAGG TCATATCATA AGGTCATATT TGAGCTTTTT 822
EgrNAC61pro reference  AAATTCCTTA TTTTTGTAAC TTACAAAAGG TCATATCATA AGGTCATATT TGAGCTTTTT 836
                                                         860                           880                           900
EgrNAC61pro cloned     TAAAAAGAAA AAAATAAGTG GCCAACTTGT CAATACCAAT CAACAGGAAC TATAACTTAG 882
EgrNAC61pro reference  TAAAAAGAAA AAAATAAGTG GCCAACTTGT CAATACCAAT CAACAGGAAC TATAACTTAG 896
                                                         920                           940                           960
EgrNAC61pro cloned     TCAACATATT CTAATTAGAT GCTTCATGGC TTACTCAATA TAACAATGCT GAACTTATTA 942
EgrNAC61pro reference  TCAACATATT CTAATTAGAT GCTTCATGGC TTACTCAATA TAACAATGCT GAACTTATTA 956
                                                         980                         1,000                         1,020
EgrNAC61pro cloned     AGACTAGTTT ACAAAATTGA TTTATAAAAT TGTCAGACAG CAAATGGCCT TTTAAACAAC 1002
EgrNAC61pro reference  AGACTAGTTT ACAAAATTGA TTTATAAAAT TGTCAGACAG CAAATGGCCT TTTAAACAAC 1016
                                                       1,040                         1,060                         1,080
EgrNAC61pro cloned     TTTACCAAAA AAAAAA---- ---------- ---------- ---------A TGGCCTTTTA 1029
EgrNAC61pro reference  TTTACCAAAA AAAAAATGGC CTTTTAAACA ACTTTACCAA AAAAAAAAAA TGGCCTTTTA 1076
                                                       1,100                         1,120                         1,140
EgrNAC61pro cloned     AACAAATGAT CTATAAGCAA TGACCCTTAA GTAAACGTGT AAAGAACTAA AACTATACAT 1089
EgrNAC61pro reference  AACAAATGAT CTATAAGCAA TGACCCTTAA GTAAACGTGT AAAGAACTAA AACTATACAT 1136
                                                       1,160                         1,180                         1,200
EgrNAC61pro cloned     TTTATGTATT ACTAAATGGT GTTGAAAATA GAAAGAATCG CATCTTACCA AACATGACTA 1149
EgrNAC61pro reference  TTTATGTATT ACTAAATGGT GTTGAAAATA GAAAGAATCG CATCTTACCA AACATGACCA 1196
                                                       1,220                         1,240                         1,260
EgrNAC61pro cloned     TTGATTTTCT ATTTTTGAAT GTTTTCCTGA GCAAAATGAG GAGCAAACCA AACATTTGAC 1209
EgrNAC61pro reference  TTGATTTTCT ATTTTTGAAT GTTTTCCTGA GCAAAATGAG GAGCAAACCA AACATTTGAC 1256
                                                       1,280                         1,300                         1,320
EgrNAC61pro cloned     ACTATAAGTG TTATTTTGAC TTAACCTTGA GTCAAAACTA ATTAATGTTA ACAAATGGAT 1269
EgrNAC61pro reference  ACTATAAGTG TTATTTTGAC TTAACCTTGA GTCAAAACTA ATTAATGTTA ACAAATGGAT 1316
                                                       1,340                         1,360                         1,380
EgrNAC61pro cloned     TTGATCTTGT TAGAATGCTA ATGACTAAAT ATTTAGATTC GCAACATTTA ATTAAGTCAT 1329
EgrNAC61pro reference  TTGATCTTGT TAGAATGCTA ATGACTAAAT ATTTAGATTC GCAACATTTA ATTAAGTCAT 1376
                                                       1,400                         1,420                         1,440
EgrNAC61pro cloned     CAAAAGAGGC TCCTCACTCG ATCAATTTAG TCATGATAGC TATGCAAAGG ATAAGTCTTC 1389
EgrNAC61pro reference  CAAAAGAGGC TCCTCACTCG ATCAATTTAG TCATGATAGC TATGCAAAAG ATAAGTCTTC 1436
                                                       1,460                         1,480                         1,500
EgrNAC61pro cloned     TTATGCAAAT TATTATTAGG GGCATTCCTT TTAATAGACT TAACCATTCG AACTTAAAAA 1449
EgrNAC61pro reference  TTATGCAAAT TATTATTAGG GGCATTCCTT TTAATAGACT TAACCATTCG AACTTAAAAA 1496
```

```
                                                    1,520                                1,540                                1,560
                                                      |                                    |                                    |
EgrNAC61pro cloned     GACAAGGATC  GCGTCCAACA  ATACCAAAAG  CTAATACATA  CGATTCTACT  GATTTTCTTT  1509
EgrNAC61pro reference  GACAAGGATC  GCGTCCAACA  ATACCAAAAG  CTAATACATA  CGATTCTACT  GATTTTCTTT  1556
                                                    1,580                                1,600                                1,620
                                                      |                                    |                                    |
EgrNAC61pro cloned     AAATGTGATT  GACCTAATCT  AACTTGCAAA  AATCTCCTCC  CCCACCCCCC  CTCCCCACCC  1569
EgrNAC61pro reference  AAATGTGATT  GACCTAATCT  AACTTGCAAA  AATCTCCTCC  CCCACCCCCC  CTCCCCACCC  1616
                                                    1,640                                1,660                                1,680
                                                      |                                    |                                    |
EgrNAC61pro cloned     TCTCTCTCGC  TAGGTGGTTG  GCTATTGCGG  GAGCTCAGAC  CCCACCTTAA  GTACACAGTA  1629
EgrNAC61pro reference  TCTCTCTCGC  TAGGTGGTTG  GCTATTGCGG  GAGCTCAGAC  CCCACCTTAA  GTACACAGTA  1676
                                                    1,700                                1,720                                1,740
                                                      |                                    |                                    |
EgrNAC61pro cloned     GTGAACAATC  ATATTCTAAC  TTTTCATGCA  ACTTTGGACC  TACATGTGTC  CTACCACCAT  1689
EgrNAC61pro reference  GTGAACAATC  ATATTCTAAC  TTTTCATGCA  ACTTTGGACC  TACATGTGTC  CTACCACCAT  1736
                                                    1,760                                1,780                                1,800
                                                      |                                    |                                    |
EgrNAC61pro cloned     TATAAATTAT  CATGGCTGCA  TTACCCTTTA  CGCACAGCCC  CCCGCCTCCT  CTCTCTCTCT  1749
EgrNAC61pro reference  TATAAATTAT  CATGGCTGCA  TTACCCTTTA  CGCACAGCCC  CCCGCCTCCT  CTCTCTCTCT  1796
                                                    1,820                                1,840                                1,860
                                                      |                                    |                                    |
EgrNAC61pro cloned     CTCTCTCTCT  CTCTCTCTCT  CTCTCTCCGG  CGGTTCATCT  CTCTTCCATC  TCAAAGGACG  1809
EgrNAC61pro reference  CTCTCTCTCT  CTCTCTCTCT  CTC----CGG  CGGTTCATCT  CTCTTCCATC  TCAAAGGACG  1852
                                                    1,880                                1,900                                1,920
                                                      |                                    |                                    |
EgrNAC61pro cloned     AGTTTTTCGG  TTTTTATCTT  CCTTCAGGTC  TTTTCCTATG  GAGTTCCTCA  TATAGATCAA  1869
EgrNAC61pro reference  AGTTTTTCGG  TTTTTATCTT  CCTTCAGGTC  TTTTCCTATG  GAGTTCCTCA  TATAGATCAA  1912
                                                    1,940                                1,960                                1,980
                                                      |                                    |                                    |
EgrNAC61pro cloned     AAATCTCCAC  ACACACACAC  ACACAACGCT  TTCCACTCGA  CACGCTGAGC  ATACAGCTCG  1929
EgrNAC61pro reference  AAATCTCCAC  ACACACACAC  ACACAACGCT  TTCCACTCGA  CACGCTGAGC  ATACAGCTCG  1972
                                                    2,000
                                                      |
EgrNAC61pro cloned     TTAATTCGGT  CCTCTGTTGT  AAGAGAAA  1957
EgrNAC61pro reference  TTAATTCGGT  CCTCTGTTGT  AAGAGAAA  2000
```

Figure S2. Alignment of the cloned *PagWND1Apro* sequence against the *P. grandidentata* allele (*PgWND1Apro* reference) (http://popgenie.org)

```
                                                          20                    40                    60
PgWNDA1pro reference  AAATTTAATT ATAACATGTA CTTGTAATGA CAATTTATTC AAAATATCGA TATAACCATC 60
 PagWND1Apro cloned   AAATTTAATT ATAACATGTA CTTGTAATGA CAATTTATTC AAAATATCGA TATAACCATC 60
                                                          80                   100                   120
PgWNDA1pro reference  AAACAATTGC TGCTAAATTT TCATTTTGTA TTAATGTCAC TAGATAACAG TAAATTAATT 120
 PagWND1Apro cloned   AAACAATTGC TGCTAAATTT TCATTTTGTA TTAATGTCAC TAGATAACAG TAAATTAATT 120
                                                         140                   160                   180
PgWNDA1pro reference  TATACCAATA TAAATTGTAA ACTATCAATA TCACCTGGAA TAAATTAATA ATATATACAA 180
 PagWND1Apro cloned   TATACCAATA TAAATTGTAA ACTATCAATA TCACCTGGAA TAAATTAATA ATATATACAA 180
                                                         200                   220                   240
PgWNDA1pro reference  TTAATAATGA AACATCCACC ATAAAATATA AATTATTTGA ATGAATCTCA AACTCAACTA 240
 PagWND1Apro cloned   TTAATAATGA AACATCCACC ATAAAATATA AATTATTTGA ATGAATCTCA AACTCAACTA 240
                                                         260                   280                   300
PgWNDA1pro reference  TTTGATATTA TTTGGGCCTA TTTGTTTTT- ---------T ATTTTAAAAG GGTTTTTAAA 290
 PagWND1Apro cloned   TTTGATATTA TTTGGGCCTA TTTGTTTTTA TTTGTTTTTT ATTTTAAAAG GGTTTTTAAA 300
                                                         320                   340                   360
PgWNDA1pro reference  AAAATTAAAA AAAAAATTCT TTACTTTTAA TTAATATTTT TTTTAATGTT TTCATATAAT 350
 PagWND1Apro cloned   AAAATTAAAA AAAAAATTCT TTACTTTTAA TTAATATTTT TTTTAATGTT TTCATATAAT 360
                                                         380                   400                   420
PgWNDA1pro reference  TTTAATGTGC TAATATTAAA AATAATTTTT AAATATAAAA AATATTATTA TTATGAATTT 410
 PagWND1Apro cloned   TTTAATGTGC TAATATTAAA AATAATTTTT AAATATAAAA AATATTATTA TTATGAATTT 420
                                                         440                   460                   480
PgWNDA1pro reference  CTAAACAAAA AATAATTTTA AAAACAACTA CTATATCATA TTTCCAAATA CCTTCTTACA 470
 PagWND1Apro cloned   CTAGACAAAA AATAATTTTA AAAACAACTA CTATATCATA TTTCCAAATA CCTTCTTACA 480
                                                         500                   520                   540
PgWNDA1pro reference  GCAAAGCAGC AATTAATAGC CAATGGATCA ATAAAGATCC TAGAGCTGAA CTCATTTTTA 530
 PagWND1Apro cloned   GCAAAGCAGC AATTAATAGC CAATGGATCA ATAAAGATCC TAGAGCTGAA CTCATTTTTA 540
                                                         560                   580                   600
PgWNDA1pro reference  AAATACCCAG AGATATATAT TGCAAGGGGG TAAAAATATT AAAATCACAA GTACAATATG 590
 PagWND1Apro cloned   AAATACCCAG AGATATATAT TGCAAGGGGG TAAAAATATT AAAATCACAA GTACAATATG 600
                                                         620                   640                   660
PgWNDA1pro reference  GTATAAAAAA CAATATAAAA TAAAATACAA AGGTGAAAAT ATATCATCAA TTCAAACTCA 650
 PagWND1Apro cloned   GTATAAAAAA CAATATAAAA TAAAATACAA AGGTGAAAAT ATATCATCAA TTCAAACTCA 660
                                                         680                   700                   720
PgWNDA1pro reference  ATCATCAACT TTGATCATAC TATACAGGCT TCAAAGATGC TATACTGCAT GAAACCGGAT 710
 PagWND1Apro cloned   ATCATCAACT TTGATCATAC TATACAGGCT TCAAAGATGC TATACTGCAT GAAACCGGAT 720
                                                         740                   760                   780
PgWNDA1pro reference  AGACAGTTTC TATCTTCCAA TCGATAAAAA GGGGGAAAAG AAGAAAAAAT GGGAAAGCTT 770
 PagWND1Apro cloned   AGACAGTTTC TATCTTCCAA TCGATAAAAA GGGGGAAAAG AAGAAAAAAT GGGAAAGCTT 780
                                                         800                   820                   840
PgWNDA1pro reference  TATTGTCCTC CTCTCTCACC AATTCTTTCT CAGTGGTTGC GATCTTGACT CAAGAGGAAA 830
 PagWND1Apro cloned   TATTGTCCTC CTCTCTCACC AATTCTTTCT CAGTGGTTGC GATCTTGACT CAAGAGGAAA 840
                                                         860                   880                   900
PgWNDA1pro reference  ATTGTATCTT CTTTTATCAA CGAAAGATTC AGCAGTTGCC ATGTACAATC ATGGAAAGGG 890
 PagWND1Apro cloned   ATTGTATCTT CTTTTATCAA CGAAAGATTC AGCAGTTGCC ATGTACAATC ATGGAAAGGG 900
                                                         920                   940                   960
PgWNDA1pro reference  AAGATATAAT AAGTTCAAGA AAGGAAAATG CAGATCTACA AGAGAGTCCT CTCTCACCAT 950
 PagWND1Apro cloned   AAGATATAAT AAGTTCAAGA AAGGAAAATG CAGATCTACA AGAGAGTCCT CTCTCACCAT 960
                                                         980                 1,000                 1,020
PgWNDA1pro reference  TTTGTGGGTA CATGACCATA TTCTGTAGGA CTGCTCTCCT TGGACTCTAG TAGCCATTCT 1010
 PagWND1Apro cloned   TTTGTGGGTA CATGACCATA TTCTGTAGGA CTGCTCTCCT TGGACTCTAG TAGCCATTCT 1020
                                                       1,040                 1,060                 1,080
PgWNDA1pro reference  TATTAAGGTT TTCCAAGGCA CGTGTTTGTA AATTATAATA TATATACGGA GACTGGTCTT 1070
 PagWND1Apro cloned   TATTAAGGTT TTCCAAGGCA CGTGTTTGTA AATTATAATA TATATACGGA GACTGGTCTT 1080
                                                       1,100                 1,120                 1,140
PgWNDA1pro reference  GATATATGTA GTACCATGCA TTTGCACTAG TAAAAATGTT TTCTTAAGGG CACCTTTTGT 1130
 PagWND1Apro cloned   GATATATGTA GTACCATGCA TTTGCACTAG TAAAAATGTT TTCTTAAGGG CACCTTTTGT 1140
                                                       1,160                 1,180                 1,200
PgWNDA1pro reference  TTGTATTAAT TTTGGATAAT CTGTAGTCTC CCGTTGGAGC AAAATATTGA TCACTCTCAT 1190
 PagWND1Apro cloned   TTGTATTAAT TTTGGATAAT CTGTAGTCTC CCGTTGGAGC AAAATATTGA TCACTCTCAT 1200
                                                       1,220                 1,240                 1,260
PgWNDA1pro reference  GTTATTCTAA TGCTAGCTAG TACCATGATG TGCACAGTTG GTGTCTACAA TGATAAAATG 1250
 PagWND1Apro cloned   GTTATTCTAA TGCTAGCTAG TACCATGATG TGCACAGTTG GTGTCTACAA TGATAAAATG 1260
                                                       1,280                 1,300                 1,320
PgWNDA1pro reference  TGAATAAAGA AGTGAAAGGT AGGGAGAAAT ATAGAAATTA CTCAGTCTCC ACAAAGCCCA 1310
 PagWND1Apro cloned   TGAATAAAGA AGTGAAAGGT AGGGAGAAAT ATAGAAATTA CTCAGTCTCC ACAAAGCCCA 1320
                                                       1,340                 1,360                 1,380
PgWNDA1pro reference  TCATGTCTTT CTCCCTCTAG GGACCATGAA AGGTCAGTGC CTCCCGACCC CATCATCTCT 1370
 PagWND1Apro cloned   TCATGTCTTT CTCCCTCTAG GGACCATGAA AGGTCAGTGC CTCCCGACCC CATCATCTCT 1380
                                                       1,400                 1,420                 1,440
PgWNDA1pro reference  CTCTCTTGCC CTCTTGTCGT TGAGGTGGTT GCACAAGTAA AACGAGTGAT GTTTAGAGAG 1430
 PagWND1Apro cloned   CTCTCTTGCC CTCTTGTCGT TGAGGTGGTT GCACAAGTAA AACGAGTGAT GTTTAGAGAG 1440
                                                       1,460                 1,480                 1,500
PgWNDA1pro reference  AAGAGGAGGT AGAGGGAGAA AAGGAGAGAG ATTGAGATTT TTTTCTTAGC ATAGTGGTGG 1490
 PagWND1Apro cloned   AAGAGGAGGT AGAGGGAGAA AAGGAGAGAG ATTGAGATTT TTTTCTTAGC ATAGTGGTGG 1500
```

```
                                           1,520                                   1,540                                   1,560
                                               |                                       |                                       |
PgWNDA1pro reference  TTTCAGTGGT GTTTTTCAAA TGGATATTCA GGTCATGGGT CGTAGTTGAG ATGGAGGCGT 1550
  PagWND1Apro cloned  TTTCAGTGGT GTTTTTCAAA TGGATATTCA GGTCATGGGT CGTAGTTGAG ATGGAGGCGT 1560
                                           1,580                                   1,600                                   1,620
                                               |                                       |                                       |
PgWNDA1pro reference  GCCCCCTGCA CATACAAATA CAAATAGTTA CCTCTAACGC TTCTTCTTCT TTTTTTTCTT 1610
  PagWND1Apro cloned  GCCCCCTGCA CATACAAATA CAAATAGTTA CCTCTAACGC TTCTTCTTCT TTTTTTTCTT 1620
                                           1,640                                   1,660                                   1,680
                                               |                                       |                                       |
PgWNDA1pro reference  TTTTTTTTCT TTTCTTGATG GACCCGATTG GCCAATTGTT AGTACCTATA TTGTGGGCCT 1670
  PagWND1Apro cloned  TTTTTTTTCT TTTCTTGATG GACCCGATTG GCCAATTGTT AGTACCTATA TTGTGGGCCT 1680
                                           1,700                                   1,720                                   1,740
                                               |                                       |                                       |
PgWNDA1pro reference  GGAAAAACCC TACCCTCTTT TCTATCTTTA GTGACTAGTC TCTACTTCTA GGTGGTTGGC 1730
  PagWND1Apro cloned  GGAAAAACCC TACCCTCTTT TCTATCTTTA GTGACTAGTC TCTACTTCTA GGTGGTTGGC 1740
                                           1,760                                   1,780                                   1,800
                                               |                                       |                                       |
PgWNDA1pro reference  TCTTCTCCAA GGCTTAGACC CCACAGTAAG TACACTAATG GAATTGATTC CAAAACTTTT 1790
  PagWND1Apro cloned  TCTTCTCCAA GGCTTAGACC CCACAGTAAG TACACTAATG GAATTGATTC CAAAACTTTT 1800
                                           1,820                                   1,840                                   1,860
                                               |                                       |                                       |
PgWNDA1pro reference  CATGCATTTT TGGACCCTTC CCCCACATGC CCTACAAACC ATTATAAATT ATCAGGACTG 1850
  PagWND1Apro cloned  CATGCATTTT TGGACCCTTC CCCCACATGC CCTACAAACC ATTATAAATT ATCAGGACTG 1860
                                           1,880                                   1,900                                   1,920
                                               |                                       |                                       |
PgWNDA1pro reference  CATTACCCTT TACGCACCCT CTCTCCTTGT TAATGTTGTC TCTCATTTTT GCTTCTCTTT 1910
  PagWND1Apro cloned  CATTACCCTT TACGCACCCT CTCTCCTTGT TAATGTTGTC TCTCATTTTT GCTTCTCTTT 1920
                                           1,940                                   1,960                                   1,980
                                               |                                       |                                       |
PgWNDA1pro reference  CATATTGATC TCTAGCTATT CTCTTTTTCT CTCTATCATC AGCTTTTACC TGTACGTTGA 1970
  PagWND1Apro cloned  CATATTGATC TCTAGCTATT CTCTTTTTCT CTCTATCATC AGCTTTTACC TGTACGTTGA 1980
                                           2,000                                   2,020                                   2,040
                                               |                                       |                                       |
PgWNDA1pro reference  TCATCTATCT ATATATACAC ATATATTCAC TCACTAGCCA GCTTTATACA ATAATTTGTC 2030
  PagWND1Apro cloned  TCATCTATCT ATATATACAC ATATATTCAC TCACTAGCCA GCTTTATACA ATAATTTGTC 2040

PgWNDA1pro reference  CTAGCAAAA 2039
  PagWND1Apro cloned  CTAGCAAAA 2049
```
